# Corrigendum: ‘Genome sequencing and multifaceted taxonomic analysis of novel strains of violacein-producing bacteria and non-violacein-producing close relatives’

**DOI:** 10.1099/mgen.0.001204

**Published:** 2024-02-09

**Authors:** Marina Estella De León, Harriet S. Wilson, Guillaume Jospin, Jonathan A. Eisen

**Affiliations:** ^1^​ Microbiology Graduate Group, University of California, Davis, CA, USA; ^2^​ Department of Biological Sciences, Sierra College, Rocklin, CA, USA; ^3^​ Department of Evolution and Ecology, University of California, Davis, CA, USA; ^4^​ Genome Center, University of California, Davis, CA, USA; ^5^​ AnimalBiome, Oakland, CA, USA; ^6^​ Department of Medical Microbiology and Immunology, University of California, Davis, CA, USA

## Full-Text

In the published version of the article there were etymological errors incorporated into three of the proposed species names. In addition, the format of the species descriptions did not comply with Rules 27 and 30 of the ICNP. These errors have been corrected here.

The authors apologize for any inconvenience caused by these errors.

The five novel species proposed and their descriptions are provided below:

## Description of *Aquitalea aquatica* sp. nov.

*Aquitalea aquatica* (a.qua’ti.ca. L. fem. adj. *aquatica*, aquatic, referring to the isolation source).

Cells are motile, Gram-negative (KOH-positive), non-sporing, fermentative bacilli, 0.5–1.0×1.5–2.5 µm. Colonies on tryptic soy agar (TSA) are circular, entire, low-convex, smooth-shiny, semi-opaque, milky-tan, 1–3 mm in diameter after 48 hours. Colonies on MacConkey’s (MAC) and Tergitol-7 (T-7) agar were more translucent and smaller. Positive for catalase, oxidase, citrate utilization, urea hydrolysis, the reduction of nitrate to nitrite (but not to nitrogenous gasses) and haemolysis on 5 % sheep blood. Negative for violacein formation, aesculin, starch and gelatin hydrolysis, indole and hydrogen sulfide formation on sulfide, indole, motility medium (SIM) plus the formation of acid and acetoin in methyl-red Voges-Proskauer (MR-VP) medium. Acid formed aerobically from inositol, but not from arabinose, lactose, maltose, mannitol, raffinose, rhamnose, sorbitol, sucrose or xylose; acid formed through the fermentation of glucose in oxidation/fermentation (O/F) medium. Substrates assimilated as carbon sources include pyruvic acid methylester, tween 40, tween 80, d-cellobiose, alpha-d-lactose, beta-methyl-d-glucoside, d-xylose, l-erythritol (meso erythritol), d-mannitol, N-acetyl-d-glucosamine, d-glucosaminic acid, Glucose-1-phosphate, D, l-alpha-glycerol phosphate, d-galactonic acid gamma-lactone, d-galacturonic acid, gamma-hydroxybutyric acid, alpha-ketobutyric acid, d-malic acid, l-arginine, l-asparagine, l-phenylalanine, l-serine, l-threonine, glycyl-l-glutamic acid and putrescine, but not alpha-cyclodextrin, glycogen, 2-hydroxybenzoic acid, 4-hydroxybenzoic acid, itaconic acid or phenylethylamine.

The type strain, HSC-21Su07^T^ (= DSM 114499^T^ = NRRL B-65646^T^), was isolated from spring water in northern California. The genomic G+C content of the type strain is 59.6 % and its approximate genome size is 4.45 Mbp. The GenBank/DDBJ/ENA accession numbers for the 16S rRNA gene sequence and draft genome are ON013926 and JACERN000000000, respectively.

## Description of *Chromobacterium fluminis* sp. nov.

*Chromobacterium fluminis* (flu’mi.nis. L. gen. n. *fluminis*, of a river, referring to the isolation source).

Cells are motile, Gram-negative (KOH-positive), non-sporing, fermentative bacilli, 0.7–1×2–5 µm. Colonies on tryptic soy agar (TSA) are circular, entire, raised, smooth-shiny, semi-opaque, pale pinkish-cream, 1–3 mm in diameter after 48 hours. Colony surfaces become wrinkled with age. Colonies on MAC and T-7 agar are smaller and more translucent. Positive for catalase, oxidase, citrate utilization, aesculin and gelatin hydrolysis, reduction of nitrate to nitrite (but not to nitrogenous gasses) plus beta-haemolysis on 5 % sheep blood. Negative for violacein formation, urea and starch hydrolysis, indole and hydrogen sulfide formation (SIM) plus formation of acid and acetoin in MR-VP medium. Acid formed aerobically from inositol but not from arabinose, lactose, maltose, mannitol, raffinose, rhamnose, sorbitol, sucrose or xylose; acid formed through the fermentation of glucose in O/F medium. Substrates assimilated as carbon sources include pyruvic acid methylester, tween 40, tween 80, glycogen, d-cellobiose, N-acetyl-d-glucosamine, glucose-1-phosphate, D, l-alpha glycerol phosphate, gamma-hydroxybutyric acid, alpha-ketobutyric acid, d-malic acid, l-arginine, l-asparagine, l-phenylalanine, l-serine, l-threonine, glycyl-l-glutamic acid and putrescine, but not alpha-cyclodextrin, alpha-d-lactose, beta-methyl-d-glucoside, d-xylose, l-erythritol (meso-erythritol), d-mannitol, d-glucosaminic acid, d-galactonic acid gamma-lactone, d-galacturonic acid, 2-hydroxybenzoic acid, 4-hydroxybenzoic acid, itaconic acid or phenylethylamine.

The type strain, HSC-31F16^T^ (= DSM 114492^T^ = NRRL B-65644^T^), was isolated from a major river in northern California. The genomic G+C content of the type strain is 62.32% and its approximate genome size is 5.70 Mbp. The GenBank/DDBJ/ENA accession numbers for the 16S rRNA gene sequence and draft genome are MW242637 and JAAOMA000000000, respectively.

## Description of *Duganella violaceipulchra* sp. nov.

*Duganella violaceipulchra* (vi.o.la.ce.i.pul’chra. L. masc. adj. *violaceus,* violet coloured; L. masc. adj. *pulcher,* beautiful; N.L. fem. adj. *violaceipulchra,* violet and beautiful, referring to the morphology of colonies).

Cells are motile, Gram-negative (KOH-positive), non-sporing, oxidative coccobacilli, 1.0–1.5×1.5–2 µm. Colonies on yeast extract malt agar (YEM) are circular, entire, high-convex, smooth-shiny, semi-opaque, milky-beige (becoming violet with age), 1–3 mm in diameter after 48 hours. Colonies become larger and rubbery over time. Positive for violacein formation, catalase, oxidase, gelatin liquefaction, starch hydrolysis and the reduction of nitrate to nitrite (but not to nitrogenous gasses). Negative for citrate utilization, urea and aesculin hydrolysis, indole and hydrogen sulfide production (SIM) and the formation of acid and acetoin from glucose fermentation (MR-VP). Not haemolytic on sheep blood agar and does not form acid aerobically from arabinose, inositol, lactose, maltose, mannitol, raffinose, rhamnose, sorbitol, sucrose or xylose in O/F medium. Substrates assimilated as carbon sources include tween 40, tween 80, alpha-cyclodextrin, glycogen, alpha-d-lactose, N-acetyl-d-glucosamine and d-galacturonic acid, but not pyruvic acid methylester, d-cellobiose, beta-methyl-d-glucoside, d-xylose, l-erythritol (meso-erythritol), d-mannitol, d-glucosaminic acid, glucose-1-phosphate, D, l-alpha-glycerol phosphate, d-galactonic acid gamma-lactone, 2-hydroxybenzoic acid, 4-hydroxybenzoic acid, gamma-hydroxybutyric acid, itaconic acid, alpha-ketobutyric acid, d-malic acid, l-arginine, l-asparagine, l-phenylalanine, l-serine, l-threonine, glycyl-l-glutamic acid, phenylethylamine or putrescine.

The type strain, HSC-15S17^T^ (= ATCC TSD-229^T^ = CCOS 2056^T^), was isolated from water dripping from a fern plant in northern California. The genomic G+C content of the type strain is 64.09 % and its approximate genome size is 7.47 Mbp. The GenBank/DDBJ/ENA accession numbers for the 16S rRNA gene sequence and draft genome are MT579559 and JAHTGR000000000, respectively.

## Description of *Iodobacter violaceini* sp. nov.

*Iodobacter violaceini* (vi.o.la.ce.i'ni. N.L. neut. n. *violaceinum*, violacein; N.L. gen. n. *violaceini*, pertaining to violacein and the morphology of colonies).

Cells are motile, Gram-negative (KOH-positive), non-sporing, fermentative bacilli, 0.7–1.0×3–5 µm. Colonies on nutrient agar (NA) are circular, entire, flat, smooth-shiny, opaque to semi-translucent, violet and 2–4 mm in diameter after 48. Evidence of swarming appeared over time. Positive for violacein formation, catalase, gelatin hydrolysis, reduction of nitrate to nitrite (but not to nitrogenous gasses) and beta-haemolysis on 5 % sheep blood. Negative for oxidase, citrate utilization, urea, starch and aesculin hydrolysis, indole and hydrogen sulfide formation (SIM) and the formation of acid and acetoin in MR-VP medium. Acid formed aerobically from maltose but not from arabinose, inositol, lactose, mannitol, raffinose, rhamnose, sorbitol, sucrose or xylose; acid formed through the fermentation of glucose in O/F medium. Substrates assimilated as carbon sources include pyruvic acid methylester, tween 40, N-acetyl-d-glucosamine, glucose-1-phosphate, l-threonine and glycyl-l-glutamic acid, but not tween 80, alpha-cyclodextrin, glycogen, d-cellobiose, alpha-d-lactose, beta-methyl-d-glucoside, d-xylose, l-erythritol (meso-erythritol), d-mannitol, d-glucosaminic acid, D, l-alpha-glycerol phosphate, d-galactonic acid gamma-lactone, d-galacturonic acid, 2-hydroxybenzoic acid, 4-hydroxybenzoic acid, gamma-hydroxybutyric acid, itaconic acid, alpha-ketobutyric acid, d-malic acid, l-arginine, l-asparagine, l-phenylalanine, l-serine, phenylethylamine or putrescine.

The type strain, HSC-16F04^T^ (= DSM 114489^T^ = NRRL B-65647^T^), was isolated from a hand-dug well in northern California. The genomic G+C content of the type strain is 49.41 % and its approximate genome size is 4.77 Mbp. The GenBank/DDBJ/ENA accession numbers for the 16S rRNA gene sequence and draft genome are ON013928 and JAAOLX000000000, respectively.

## Description of *Massilia hydrophila* sp. nov.

*Massilia hydrophila* (hy.dro’phi.la. Gr. neut. n. *hydro*, water; N.L. fem. adj. *philus* [from Gr. masc. adj. *philos*], loving; N.L. fem. adj. *hydrophila*, water loving, referring to the isolation source).

Cells are motile, Gram-negative (KOH-positive), non-sporing, oxidative bacilli, typically 0.7–1.0×2–5 µm (some reached 20 µm in length). Colonies on tryptic soy agar (TSA) are circular, entire, low-convex, smooth-shiny, semi-opaque, milky-beige and 1–3 mm in diameter after 48 h. Positive for catalase, oxidase, gelatin liquefaction, starch hydrolysis, the reduction of nitrate to nitrite (but not to nitrogenous gasses) and haemolysis on sheep blood agar. Negative for violacein formation, citrate utilization, urea and aesculin hydrolysis, indole and hydrogen sulfide production (SIM) and the formation of acid and acetoin from glucose fermentation (MR-VP). Acid not formed aerobically from arabinose, inositol, lactose, maltose, mannitol, raffinose, rhamnose, sorbitol, sucrose or xylose in O/F medium. Substrates assimilated as carbon sources include pyruvic acid methylester, tween 40, alpha-cyclodextrin, glycogen, l-asparagine, l-serine, l-threonine and glycyl-l-glutamic acid as single carbon sources, but not tween 80, d-cellobiose, alpha-d-lactose, beta-methyl-d-glucoside, d-xylose, l-erythritol (meso-erythritol), d-mannitol, N-acetyl-d-glucosamine, d-glucosaminic acid, glucose-1-phosphate, D, l-alpha-glycerol phosphate, d-galactonic acid gamma-lactone, d-galacturonic acid, 2-hydroxybenzoic acid, 4-hydroxybenzoic acid, gamma-hydroxybutyric acid, itaconic acid, alpha-ketobutyric acid, d-malic acid, l-arginine, l-phenylalanine, phenylethylamine or putrescine.

The type strain, HSC-2F05^T^ (= DSM 114498^T^ = NRRL B-65648^T^), was isolated from a freshwater pond in northern California. The genomic G+C content of the type strain is 66.7 % and its approximate genome size is 4.68 Mbp. The GenBank/DDBJ/ENA accession numbers for the 16S rRNA gene sequence and draft genome are ON013927 and JAHYBX000000000, respectively.

